# Antimalarial activity of a *cis*-terpenone

**DOI:** 10.1186/1475-2875-8-139

**Published:** 2009-06-25

**Authors:** DC Ghislaine Mayer, Maimuna Bruce, Olga Kochurova, Jennifer K Stewart, Qibing Zhou

**Affiliations:** 1 Department of Biology, Virginia Commonwealth University, 1000 W. Cary St, Richmond, Virginia, 23284-2012, USA; 2 Department of Chemistry, Virginia Commonwealth University, 1001 W. Main St, Richmond, Virginia, 23284-2006, USA

## Abstract

**Background:**

Malaria is the third most prevalent cause of infectious disease in the world. Resistance of the parasite to classical drugs makes the discovery of new and effective drugs more urgent. The oxidized derivative of hydroxy-*cis *terpenone (OHCT) is a synthetic molecule that is not toxic to cultured human liver cells at concentrations as high as 60 μM and inhibits activity of cytochrome P450s that metabolize many drugs.

**Methods:**

OHCT activity against chloroquine-sensitive and -resistant strains of *Plasmodium falciparum*, and a *P. falciparum *clone that is partially resistant to artemisinin was assayed *in vitro.*

**Results:**

OHCT at nanomolar concentrations was effective against all intraerythrocytic stages of *P. falciparum *and exhibited activity *in vitro *against both chloroquine-sensitive and -resistant strains of *P. falciparum *as well as a *P. falciparum *clone that is partially resistant to artemisinin. Moreover, OHCT exhibited potent activity against gametocytes, the form that is transmitted by mosquitoes and essential for the spread of malaria.

**Conclusion:**

OHCT displays strong growth inhibitory activity against all stages of *P. falciparum *and no evidence of toxicity to human cells in culture. It is easily synthesized and has the potential for inhibiting metabolism of drugs used in combination therapies.

## Background

Anti-malarial drug resistance is the major challenge to reducing mortality caused by *Plasmodium falciparum *infection. Parasite resistance has caused some of the least expensive, traditional anti-malarial drugs to be ineffective. Because there is concern that resistance will emerge against the current first-line drugs, such as the artemisinin-based combination therapy, there is currently great interest in discovering the next generation of anti-malarial drugs.

Terpenes isolated from the roots of several plant families have a broad range of biological activities, including anti-microbial and anti-plasmodial activity [1,2]. It has been shown that a number of terpenes and terpene derivatives isolated from a variety of sources ranging from plants to marine fungi kill *P. falciparum *parasites [3-6]. Synthetic *cis*-terpenones, including the oxidized derivative of hydroxy-*cis *terpenone (OHCT), are synthetic analogues of natural terpene quinone methides that have a broad spectrum of biological activities [7].

Synthesis of HCT and OHCT was described previously [7,8]. It was previously shown that OHCT protects human liver cells against aflatoxin and inhibits activity of liver microsomal cytochrome P450 3A4 [9]. This enzyme not only activates toxins, such as aflatoxin [10], but also contributes to the degradation of anti-malarials, such as artemisinin, thus suggesting that OHCT might increase the half-life of current anti-malarials [11]. Based on the anti-plasmodial effects of terpenes [1,2], the anti-malarial effects of OHCT were investigated.

## Methods

### Culture of *Plasmodium falciparum*

Four clones of *P. falciparum*, the chloroquine-sensitive clone HB3, the two chloroquine-resistant clones, FCR3-Gambia and Dd2Nm-Indochina, and the laboratory-induced artemisinin-resistant 7G6R, were cultured by a method modified from that of Trager and Jensen [12] in a 5% CO_2 _atmosphere at 37°C.

### *In vitro *selection of artemisinin-resistant parasite lines

The *Plasmodium falciparum *7G8 clone was used for the selection of artemisinin (ART) resistance. Drug resistance selection experiments were performed as previously described [13]. After the parasitaemia reached 2–3%, frozen stocks of ART-selected parasites were prepared with Glycerolyte.

### Anti-malarial *in vitro *activity

Chloroquine diphosphate, artemisinin (Sigma Aldrich), and OHCT were dissolved in medium (RPMI 1640), ethanol and DMSO, respectively. Compounds were further diluted in medium to give a final concentration of 0.1% ethanol or DMSO. Solutions were checked to determine that precipitation did not occur under these conditions. Parasite growth was estimated by microscopic observation of Giemsa-stained blood smears and the parasite lactate dehydrogenase (pLDH) activity [14]. Effective concentrations that prevented survival of parasites were determined. Unless indicated, all results presented are the means of at least three independent experiments, and each experiment was performed in duplicate. Assays were performed for 48 h at concentrations ranging from 25 nM-50 μM.

### Production of *Plasmodium falciparum *gametocytes

The *P. falciparum *Dd2Nm clone was cultured in medium supplemented with 0.5% Albumax II, and O^+ ^human erythrocytes. The culture was treated with PIGPA solution (50 mM hydrogen phosphate and 5 mM adenine in 0.9% (w/v) NaCl, pH7.2 and 50 mg/L of hypoxanthine) as previously described [15]. Gametocytes were cultured in 2% erythrocyte suspension and started with 1% parasitaemia, containing mostly ring stage trophozoite after synchronization with sorbitol treatment. The medium was replaced on days 4, 6 and 8. After sorbitol treatments on day 9, 10 and 11, the number of asexual parasites was reduced to 99%. For sorbitol treatment, 2.5 volumes of 5% (w/v) sorbitol were added once a day. Pure gametocyte cultures of *P. falciparum *Dd2Nm clone were achieved on day 11 with an average number of gametocytes of 315 and 282 per 8,500 erythrocytes. These gametocytes that were used in the drug studies were in the range of 19% stage I, 21% stage II, 39% stage III and 21% stage IV.

### Gametocytocidal effects of OHCT against *P. falciparum in vitro*

Following sorbitol treatment, an aliquot of 150 μL of a 2.0% erythrocyte suspension containing gametocytes was transferred to a 96-well plate containing 5 μL of drugs in each well at a concentration ranging from 50 μM-25 nM for another 48 h. Thin blood films were prepared and gametocytes were counted per 7,000–10,000 erythrocytes. The effect of each drug concentration was assessed in two independent experiments in duplicate. The gametocytocidal action of each drug was recorded and the IC_50 _and IC_90 _were determined.

### Time of OHCT action on the erythrocytic life cycle

*Plasmodium falciparum *cultures were synchronized with 5% sorbitol and the Percoll-Sorbitol method. Dilutions of OHCT, chloroquine, and artemisinin were prepared. After synchronization, the parasites were plated at the ring stage in 96-well plates. Cultures were treated with OHCT or vehicle for 8 hours, and parasite viability was measured by the parasite lactate dehydrogenase (pLDH) activity [14]. The cultures were centrifuged at 10,000 g (Fisher scientific), washed in incomplete media and replaced with fresh drug every hour to remove dead parasites.

## Results

### Anti-malarial activity of OHCT

The inhibitory concentration of OHCT was determined by incubating the four *P. falciparum *clones with OHCT, chloroquine and artemisinin, respectively. As shown in Table 1, OHCT inhibited survival of all *P. falciparum *clones examined with an IC_50 _ranging from 3.9 – 93 nM, and its IC_90 _was 17 – 523 nM. OHCT and chloroquine exhibited similar potency against clone Dd2Nm. Although OHCT had a lower IC_50 _than chloroquine against the chloroquine-resistant clone FCR3, it was less potent than chloroquine against the chloroquine-sensitive clone HB3. Despite selection of 7G8R parasites that could survive in artemisinin, the resistant parasites that were thawed and cultured for the drug assay were consistently killed by artemisinin at low nanomolar concentrations. It should be noted that induced resistance to artemisinin in this clone is reported to be unstable [13]. OHCT at low nanomolar concentrations also inhibited survival of the artemisinin resistant 7G8R clone. Thus, OHCT is effective against all *P. falciparum *clones examined and might work well in combination with established anti-malarials, such as artemisinin and chloroquine.

**Table 1:** Inhibition of *Plasmodium *survival by OHCT (nM)*

	**Dd2Nm**	**HB3**	**FCR3**	**78GR**
**OHCT-IC**_50_	85 ± 36.1	38 ± 9.7	93 ± 48.7	3.9 ± 0.5
**-IC**_90_	422 ± 27.4	523 ± 310	407 ± 150	17.4 ± 0.9
**Chlor-IC**_50_	85 ± 22.5		181 ± 92.8	
**-IC**_90_	546 ± 69.1	< 20	524 ± 150.3	
**Artem-IC**_50_	-	-	-	4.2 ± 0.4
**-IC**_90_	-	-	-	18.1 ± 1.0

* *P. falciparum *clones, two chloroquine resistant clones, Dd2Nm and FCR3, one chloroquine sensitive clone, HB3, and the artemisinin resistant clone 78 GR were incubated with five concentrations of OHCT for 48 h. Percent parasitaemia in 9,500 infected human erythrocytes was estimated by microscopic observation of Giemsa-stained blood smears and by measurement of p(LDH). IC_50 _and IC_90 _values (nM) represent the mean ± SE of 3 experiments (vehicle and 5 duplicate concentrations per experiment).

OHCT at 1 – 16 μM kills most parasites within 24 hours (data not shown). The time course of action of 1 μM OHCT was investigated with the *P. falciparum *clone Dd2. As shown in Table 2, the parasitaemia level decreased by 50% following treatment with OHCT for 8 h, suggesting OHCT has a fast mechanism of action.

**Table 2:** Time course of OHCT action.

**Time (min)**	**Parasitaemia (%)**
30 min	8.6
60	8.5
120	8.6
240	8.1
360	6
480	4.7
Vehicle (0.1% DMSO)	8.9
Media	9.1

The *P. falciparum *clone Dd2 (CQR) was treated with 0.5 μM OHCT for the times shown. Parasite viability was measured by the lactate dehydrogenase assay.

**Figure 1:**
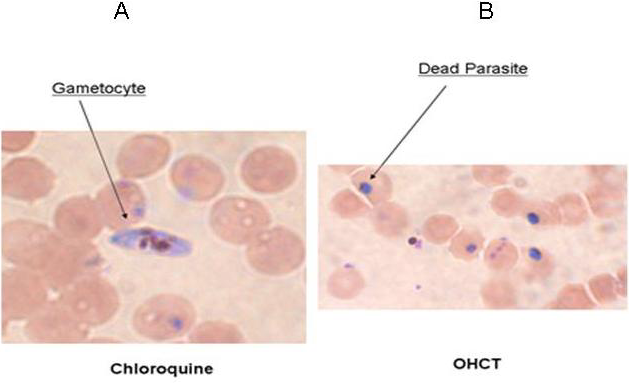
**Effect of OHCT on developmental stages of *P. falciparum *gametocytes**. Stage III gametocytes are resistant to chloroquine (A) whereas they are sensitive to OHCT (B). Gametocytes and dead parasites are shown by arrow. Representative Giemsa stain of *P. falciparum *(Dd2Nm) culture treated with chloroquine (A) and OHCT (B) at 0.5 μM for 48 h (1000×).

The *P. falciparum *clone, Dd2Nm, produces gametocytes in culture. When OHCT was tested against gametocyte cultures of *P. falciparum *Dd2Nm, decreased survival with an IC_50 _17-fold lower than the IC_50 _of chloroquine with stage I and stage II gametocytes and 26-fold lower than that of chloroquine with stage III and IV gametocytes was observed (Table 3 and Figure 1). The IC_90 _of OHCT actions on gametocytes was 9-fold lower than that of chloroquine (Table 3). These data indicate that *P. falciparum *gametocytes are more sensitive to the action of OHCT than chloroquine.

**Table 3:** OHCT (nM) inhibits growth of *P. falciparum *gametocytes*

**OHCT** **(Stage I and II)**	**Chloroquine** **(Stage I and II)**	**OHCT** **(Stage III and IV)**	**Chloroquine** **(Stage III and IV)**
**(IC_50_) **22 ± 3.4	394 ± 127.0	36 ± 9.0	944 ± 92.0
**(IC_90_) **174 ± 5.4	1520 ± 27.5	226 ± 49.3	2109 ± 85.2

*IC_50 _and IC_90 _values (nM) represent the mean ± SE of duplicate experiments, each performed in duplicate (vehicle and 8 concentrations per experiment

## Discussion

In this paper, the *in vitro *anti-malarial activity of OHCT on all blood stages of chloroquine-resistant and artemisinin-resistant *P. falciparum *clones, including the mosquito-transmissible gametocytes, is reported. OHCT displayed a significant inhibitory activity with an IC_50 _in the range of 3.9 – 93 nM depending on the parasite clone tested. OHCT is very active against the gametocyte stages of *P. falciparum*. Unlike chloroquine OHCT killed stage III gametocytes [16]. OHCT, like artemisinin, rapidly kills the early ring stage *in vitro*, suggesting that it could be used in combination with a slow-acting drug, such as chloroquine. The previous demonstrations that OHCT inhibits human liver cytochrome P450 3A4 [8,9] further suggests that it may reduce metabolism of anti-malarial compounds, such as artemisinin, that are metabolized by this enzyme [11], thus increasing the half-life and rendering them more effective. The inhibition of human liver cytochrome P450 3A4 by OHCT was shown to be mixed competitive inhibition indicating that OHCT was binding at a site different from the active site [9]. Mixed enzymatic inhibition takes place by alteration in the conformation of cytochrome P450 3A4. Thus, OHCT would not be metabolized by cytochrome P450 3A4.

OHCT is a small hydrophobic organic molecule that does not resemble any structure of known anti-malarials. In addition, OHCT and its precursor exhibit no cytotoxicity at concentrations of 10–60 μM in either human liver cells [8,9] or human lung cells (unpublished data). Both compounds are able to protect human liver cells against aflatoxin B_1_-induced toxicity [7-9]. Therefore, it is conceivable that the nanomolar anti-malarial activity of OHCT may due to a unique mechanism yet to be identified, which is currently under investigation. It is also conceivable that in combination with other known anti-malarials, OHCT would be effective *in vivo *against the drug resistant forms of the parasite.

## Conclusion

This study indicates that OHCT displays strong anti-malarial activity in the nanomolar range against *P. falciparum*. In addition, OHCT is particularly effective on the gametocyte stage. Previous studies suggest OHCT has the potential for inhibiting metabolism of drugs used in anti-malarial combination therapies and at μM concentrations exhibits no toxicity to human cells in culture.

## Competing interests

The authors declare that they have no competing interests.

## Authors' contributions

DCGM – Designed and performed *in vitro *assays; MB and OK assisted with *in vitro *assays; JKS – Contributed to over-all design of the study, performed data analyses; QZ – Designed and synthesized oxidized hydroxy *cis*-terpenone. All authors have read and approved the final manuscript.
